# Examining Geographical Differences in the HIV Care Continuum Among Men Who Have Sex with Men in Mexico

**DOI:** 10.1007/s10461-022-03809-z

**Published:** 2022-09-26

**Authors:** Angel B Algarin, Marisol Valenzuela Lara, Johanna Chapin-Bardales, Ricardo Baruch-Dominguez, Travis H Sanchez, Mauricio Hernandez-Avila, Laramie R. Smith

**Affiliations:** 1 Division of Infectious Diseases and Global Public Health, University of California San Diego, 9500 Gilman Drive, 92093-0507 La Jolla, CA USA; 2 Department of Epidemiology, Rollins School of Public Health, Atlanta, GA USA; 3 Division of HIV Prevention, Centers for Disease Control and Prevention, Atlanta, GA USA; 4 Western Hemisphere Region, International Planned Parenthood Federation, Mexico City, Mexico; 5 Economic and Social Benefits, Mexican Institute of Social Security, Mexico City, Mexico

**Keywords:** Mexico, Men who have sex with men, HIV care continuum, HIV testing

## Abstract

We analyzed data collected by the *Encuesta de Sexo Entre Hombres* study from 15,233 Mexican men who have sex with men (MSM) between May-July 2017 to examine differences in the HIV care continuum. Data were stratified into 6 geographical regions. Prevalence ratios assessed associations between region and care outcomes. Among participants never testing HIV positive (n = 13,583), 66.1% had ever been tested and 43.0% in the past year. Among HIV-positive persons (n = 1,650), 83.9% reported counseling post-diagnosis, 61.9% timely linkage to care, 42.4% timely CD4/viral load results, 38.2% timely access to antiretroviral therapy (ART), and 87.7% were currently on ART. The Ciudad de México /Estado de México region had significantly superior care continuum outcomes in ever and recent HIV testing, linkage to care, CD4/viral load results, and current ART use. Understanding geographical variations in HIV care for MSM in Mexico is one important step to inform efforts for ending HIV/AIDS by 2030 in Latin America.

## Resumen

Analizamos los datos de 15,233 hombres mexicanos que tienen sexo con hombres (HSH) recopilados entre mayo y julio de 2017 por el estudio *Encuesta de Sexo Entre Hombres* para examinar las diferencias en el continuo de la atención del VIH. Los datos se estratificaron en seis regiones geográficas. Se utilizaron razones de prevalencia para evaluar las asociaciones entre la región y los resultados de la atención. Entre los participantes sin prueba de VIH positiva (n = 13 583), el 66.1% se había hecho la prueba alguna vez en su vida y el 43.0% en el último año. Entre las personas que refirieron pruebas de VIH positivas (n = 1 650), el 83.9% informó asesoramiento post-diagnóstico, el 61.9% vinculación oportuna a la atención, el 42.4% resultados oportunos de CD4/carga viral, el 38.2% acceso oportuno a la terapia antirretroviral (TAR) y el 87.7% continuaba en TAR. La región de la Ciudad de México/Estado de México tuvo resultados del continuo de la atención significativamente superiores en pruebas de VIH pasadas y recientes, vinculación con la atención, resultados de carga viral/CD4 y uso actual de TAR. Comprender las variaciones geográficas de la atención del VIH en HSH en México es un paso importante para informar los esfuerzos para poner fin a la epidemia de HIV/Sida para 2030 en Latinoamérica.

## Introduction

Between 2010 and 2019, annual HIV infections rose by 21% in Latin America. [1] In 2017, Mexico ranked second only to Brazil in the percent of new HIV infections (14%) and AIDS-related deaths (11%) in Latin America. [2] In 2019, there were an estimated 270,000 people living with HIV (PLWH) in Mexico. [3] Among PLWH in Mexico, only an estimated 74% were aware of their HIV status, 68% were on antiretroviral therapy (ART), and 56% were virally suppressed. [3] Men who have sex with men (MSM), sex workers, people who inject drugs, transgender people, and people who are incarcerated are at highest risk for HIV acquisition in Mexico. [3] HIV prevalence among MSM is disproportionately higher than that of the general population in Mexico (17.4% vs. 0.26%). [3, 4, 5, 6] Yet, minimal research has reported on regional HIV prevalence, testing, and care continuum outcomes specifically among MSM (HIV prevalence estimates, [3–6] yearly HIV tests used, [3, 7] HIV testing within the past 12 months [8]). To reach the Joint United Nations Programme on HIV/AIDS’s (UNAIDS) 90/90/90 goal, [9] it remains important to examine care continuum outcomes and identify regions where resources may be needed to improve these outcomes and achieve national goals.

The HIV care continuum is a framework for monitoring HIV and primarily includes: HIV diagnosis, linkage to care, retention in care, ART prescription, and viral suppression [10]; further, supporting services such as HIV counseling at diagnosis and receipt of CD4/viral load lab results are intermediates in the care continuum and remain important to measure. For MSM at risk for HIV and who previously tested HIV negative or have an unknown status, receiving an HIV test at least once a year is critical to ensuring timely engagement in the HIV or pre-exposure prophylaxis (PrEP) continuum of care. [11] Measuring gaps in successful progression through the care continuums can help healthcare providers and policy makers take action to address specific gaps in HIV services.

To date, the majority of recent HIV research among MSM in Mexico has occurred in urban centers, primarily Tijuana [12–16] and Mexico City. [17–20] Additional research examining regional variations of the HIV care continuum outcomes in Mexico [21] would shed new light on planning for future HIV intervention programs that are tailored to regional epidemiological, social, and economic contexts. Reaching key populations in both urban and rural areas remains a challenge to assessing these regional variations. Online data collection may alleviate barriers to recruitment and inclusion of a geographically diverse sample in HIV research.

In this study, we aimed to assess HIV care continuum outcomes among MSM in Mexico and describe regional differences in the cascade to inform national and regional efforts to achieve 90-90-90 goals. We also aimed to show frequencies of regional care continuum outcomes before 2015 and during 2015 onward due to the implementation of national policy in Mexico that indicated everyone living with HIV should initiate ART regardless of CD4 cell count in 2015. [22, 23]

## Methods

We conducted analyses using data collected by the *Encuesta de Sexo Entre Hombres* (ESEH) study of MSM living in Mexico between May-July 2017. [24] ESEH was an online cross-sectional study modeled after the *American Men’s Internet Survey* in the United States [25, 26] that aimed to measure outcomes related to sexual health and access to HIV testing and care services. Participants were recruited via advertisements on popular social networking applications used by MSM (referred to as online recruitment venues) including *Facebook*, *Grindr*, *Hornet*, *Twitter*, *SoyHomosensual*, and *Desastre*. Participants were provided electronic informed consent and completed an online questionnaire that included items on sociodemographics, sexual health, substance use, stigma, and clinical engagement. Eligibility criteria included identifying as cisgender man, 18 + years of age, identify as gay/bisexual or as previously having oral/anal sex with another man, and Mexican residence. All variables were self-reported by participants. Recruitment was monitored to ensure at least 1,296 valid responses were obtained from each of the 6 geographical regions in Mexico based on estimated sample size calculations. The study was approved by the Ethical Committee at the National Institute of Public Health in Mexico and institutional research review boards of Emory University and the University of California San Diego.

### Measures

Dependent measures of the HIV care continuum were dichotomized (yes/no) and included HIV testing and care outcomes. Among those who did not self-report a positive HIV test result, participants reported any history of HIV testing and HIV testing in the last year. Among those who self-reported a positive HIV test result, participants were asked if they received counseling following their HIV-positive test result, were timely linked to care (< 1 month post-testing), had timely received CD4/viral load results (< 1 month post-linkage), had timely accessed (i.e. initiated) ART (< 1 month post-CD4/viral load results), and were currently taking ART.

Independent measures were informed by the sociodemographic characteristics included in Bautista-Arredondo et al. (2013), [4] recruitment venues of the AMIS sample in Sanchez et al. (2016), [26] and the heterogeneity in effective healthcare access based on insurance in Mexico in Gutierrez et al. (2014). [17] These variables included age group (18–24, 25–29, 30–39, 40+), education level (< high school, high school degree, technical/bachelor’s degree, graduate degree), recruitment venue (Facebook, Grindr, Hornet, Twitter, SoyHomosensual, Desastre, Other), insurance (Mexican Social Security Institute [IMSS], Institute for Social Security and Services for State Workers [ISSTE], Seguro Popular (converted to the Institute of Health for Welfare [INSABI] in 2020), multiple options, other, none), and HIV status (positive, negative, unknown). For those self-reporting an HIV-positive status, we created a binary variable classifying those who received their first positive HIV test before 2015 (pre-2015) vs. during 2015 and onward (post-2015), due to the implementation of policy that indicated everyone living with HIV should initiate ART regardless of CD4 cell count in 2015. [22, 23]

Based on reported state of residence, participants were stratified into the following 6 geographical regions: *Noroeste* (Baja California, Baja California Sur, Sonora, Sinaloa, Chihuahua, and Durango); *Noreste* (Coahuila, Nuevo León, Tamaulipas, San Luis Potosí, and Zacatecas); *CDMX/EdoMex* (Ciudad de México and Estado de México); *Centro* (Hidalgo, Puebla, Tlaxcala, Morelos, Guerrero, and Veracruz); *Bajío/Occidente* (Aguascalientes, Nayarit, Jalisco, Colima, Guanajuato, Michoacán, and Querétaro); and *Sur/Sureste* (Oaxaca, Tabasco, Chiapas, Campeche, Yucatán, and Quintana Roo). As there is no national consensus for categorizing states into regions to assess HIV-related outcomes in Mexico, [3–5] we developed these categorizations based on previous literature by Bautista-Arredondo et al. (2013) [4] and applied modifications informed by consultations with representatives from the National Center of HIV/AIDS Control and Prevention in Mexico (CENSIDA; the governmental institution responsible for preventing and monitoring sexual, blood, and perinatal transmission of HIV in Mexico) and other subject matter experts.

### Analysis

Data were analyzed using SAS Studio® (SAS Institute, Cary, NC). We described the characteristics of the sample overall and by geographical region using frequencies and percentages and tested for bivariate differences using Pearson’s chi-squared tests. We obtained adjusted prevalence ratios (aPR), 95% confidence intervals (95% CI), and corresponding p-values for the associations between geographical region and HIV testing and care continuum outcomes using log-linked Poisson regression models with robust standard errors. Adjusted model covariates included online recruitment venue, age, education, insurance type, and year of diagnosis (for outcomes among HIV-positive participants). Finally, we used ArcMap (v10.8; Environmental Systems Research Institute Inc., Redlands, CA) to create a geographical depiction of the regional differences in the HIV care continuum pre- and post-2015. α was set at 0.05 for all statistical analyses.

## Results

A total of 15,875 participants were eligible and completed the survey. Of those, 642 were removed from the sample due to missingness of HIV status, leaving a final analytic sample of 15,233. Most participants resided in CDMX/EdoMex (34.2%), followed by Bajío/Occidente (18.3%), Centro (15.0%), Sur/Sureste (11.9%), Noreste (10.5%), and Noroeste (10.2%). The largest proportion of the sample was recruited on Grindr (45.2%), followed by Facebook (19.3%) and Twitter (13.2%). The majority of the sample was ≤ 29 years of age (66.3%), had a Technical/Bachelor’s degree (51.7%), received healthcare services through IMSS (44.7%), and did not self-report a positive HIV test (89.2%). Significant variations by region on all descriptive characteristics can be found in Table 1.

**Table 1:** Descriptive sample characteristics of men who have sex with men in Mexico who participated in the 2017 Encuesta de Sexo Entre Hombres study by self-reported HIV status

	Total	Noroeste* (n = 1,551)	Noreste* (n = 1,595)	CDMX/EdoMex* (n = 5,206)	Centro* (n = 2,287)	Bajío/Occidente* (n = 2,788)	Sur/Sureste* (n = 1,806)	χ^2^; p-value
	n (%)	n (%)	n (%)	n (%)	n (%)	n (%)	n (%)	
**Age**								74.1; <0.001
18–24 years	6103 (40.1%)	598 (38.6%)	631 (39.6%)	1944 (37.3%)	1018 (44.5%)	1169 (41.9%)	743 (41.1%)	
25–29 years	3994 (26.2%)	393 (25.3%)	415 (26.0%)	1353 (26.0%)	616 (26.9%)	739 (26.5%)	478 (26.5%)	
30–39 years	3721 (24.4%)	402 (25.9%)	385 (24.1%)	1354 (26.0%)	482 (21.1%)	659 (23.6%)	439 (24.3%)	
40 + years	1415 (9.3%)	158 (10.2%)	164 (10.3%)	555 (10.7%)	171 (7.5%)	221 (7.9%)	146 (8.1%)	
**Education**								52.9; <0.001
< High school	22 (0.2%)	2 (0.1%)	3 (0.2%)	5 (0.1%)	6 (0.3%)	5 (0.2%)	1 (0.1%)	
High School Degree	5270 (35.2%)	504 (33.0%)	559 (35.8%)	1814 (35.4%)	807 (35.9%)	935 (34.0%)	651 (36.7%)	
Technical/Bachelor’s degree	7756 (51.7%)	841 (55.1%)	805 (51.5%)	2533 (49.4%)	1184 (52.6%)	1461 (53.1%)	932 (52.5%)	
Graduate Degree	1945 (13.0%)	180 (11.8%)	196 (12.5%)	777 (15.1%)	252 (11.2%)	350 (12.7%)	190 (10.7%)	
**HIV Status**								83.2; <0.001
Negative or unknown	13583 (89.2%)	1427 (92.0%)	1472 (92.3%)	4520 (86.8%)	2008 (87.8%)	2558 (91.8%)	1598 (88.5%)	
Positive	1650 (10.8%)	124 (8.0%)	123 (7.7%)	686 (13.2%)	279 (12.2%)	230 (8.3%)	208 (11.5%)	
**HIV Diagnosed pre-/post-2015** ^†^								12.7; 0.027
Pre-2015	820 (51.0%)	53 (44.9%)	60 (50.8%)	373 (55.4%)	120 (44.0%)	114 (50.9%)	100 (49.3%)	
Post-2015	789 (49.0%)	65 (55.1%)	58 (49.2%)	300 (44.6%)	153 (56.0%)	110 (49.1%)	103 (50.7%)	
**Insurance Type**								308.3; <0.001
IMSS	6001 (44.7%)	648 (46.7%)	667 (48.0%)	1972 (42.6%)	808 (40.6%)	1177 (48.2%)	729 (46.3%)	
ISSTE	886 (6.6%)	119 (8.6%)	96 (6.9%)	291 (6.3%)	137 (6.9%)	123 (5.0%)	120 (7.6%)	
Seguro Popular	1609 (12.0%)	135 (9.7%)	115 (8.3%)	493 (10.7%)	352 (17.7%)	291 (11.9%)	223 (14.2%)	
Private only	570 (4.2%)	50 (3.6%)	52 (3.7%)	244 (5.3%)	65 (3.3%)	107 (4.4%)	52 (3.3%)	
Other	287 (2.1%)	54 (3.9%)	24 (1.7%)	98 (2.1%)	54 (2.7%)	20 (0.8%)	37 (2.4%)	
Multiple	2228 (16.6%)	233 (16.8%)	277 (19.9%)	865 (18.7%)	238 (12.0%)	430 (17.6%)	185 (11.8%)	
None	1832 (13.7%)	148 (10.7%)	158 (11.4%)	666 (14.4%)	338 (17.0%)	295 (12.1%)	227 (14.4%)	
**Recruitment Source**								1,234; <0.001
Facebook	2944 (19.3%)	430 (27.7%)	351 (22.0%)	551 (10.6%)	613 (26.8%)	519 (18.6%)	480 (26.6%)	
Grindr	6886 (45.2%)	716 (46.2%)	802 (50.3%)	2219 (42.6%)	989 (43.2%)	1218 (43.7%)	942 (52.2%)	
Hornet	161 (1.1%)	25 (1.6%)	9 (0.6%)	10 (0.2%)	52 (2.3%)	30 (1.1%)	35 (1.9%)	
Twitter	2015 (13.2%)	195 (12.6%)	199 (12.5%)	801 (15.4%)	258 (11.3%)	410 (14.7%)	152 (8.4%)	
SoyHomosensual	1868 (12.3%)	147 (9.5%)	166 (10.4%)	803 (15.4%)	213 (9.3%)	420 (15.1%)	119 (6.6%)	
Desastre	278 (1.8%)	15 (1.0%)	38 (2.4%)	118 (2.3%)	45 (2.0%)	38 (1.4%)	24 (1.3%)	
Other	1081 (7.1%)	23 (1.5%)	30 (1.9%)	704 (13.5%)	117 (5.1%)	153 (5.5%)	54 (3.0%)	

Abbreviations: HIV, human immunodeficiency virus; IMSS, Instituto Mexicano del Seguro Social; ISSTE, Instituto de Seguridad y Servicios Sociales de los Trabajadores del Estado; CDMX/EdoMex, Ciudad de México and Estado de México

^*^ Noroeste: Baja California, Baja California Sur, Sonora, Sinaloa, Chihuahua, and Durango; Noreste: Coahuila, Nuevo León, Tamaulipas, San Luis Potosí, and Zacatecas; CDMX/EdoMex: Ciudad de México and Estado de México; Centro: Hidalgo, Puebla, Tlaxcala, Morelos, Guerrero, and Veracruz; Bajío/Occidente: Aguascalientes, Nayarit, Jalisco, Colima, Guanajuato, Michoacán, and Querétaro; and Sur/Sureste: Oaxaca, Tabasco, Chiapas, Campeche, Yucatán, and Quintana Roo.

^†^Only among participants whose self-reported HIV status was positive.

### Findings among HIV negative/unknown participants

Of the 13,583 (89.2%) participants that reported an HIV negative or unknown status, 66.1% reported ever being tested for HIV and 43.0% reported being tested within the last year. Significant variations in ever testing for HIV and testing for HIV in the past 12 months were found by geographical region (Table 2).

**Table 2:** Frequencies and adjusted prevalence ratios of ever testing for HIV and testing for HIV in the past 12 months by region among HIV-negative/unknown status men who have sex with men in Mexico—Encuesta de Sexo Entre Hombres, 2017

Region	Ever tested			Tested in past 12 months		
	n/N^‡^ (%)	aPR† (95% CI)	p-value	n/N^‡^ (%)	aPR† (95% CI)	p-value
Noroeste	918/1427 (64.3)	**0.94** **(0.90, 0.98)**	**0.007**	543/1366 (39.8)	**0.86** **(0.79, 0.93)**	**< 0.001**
Noreste	945/1472 (64.2)	**0.94** **(0.90, 0.99)**	**0.009**	587/1408 (41.7)	**0.91** **(0.85, 0.98)**	**0.013**
CDMX/EdoMex	3152/4520 (69.7)	REF	REF	2051/4341 (47.2)	REF	REF
Centro	1270/2008 (63.2)	**0.94** **(0.90, 0.98)**	**0.004**	763/1929 (39.6)	**0.85** **(0.80, 0.92)**	**< 0.001**
Bajío/Occidente	1626/2558 (63.6)	**0.94** **(0.91, 0.98)**	**0.001**	992/2458 (40.4)	**0.88** **(0.83, 0.94)**	**< 0.001**
Sur/Sureste	1062/1598 (66.5)	0.98 (0.94, 1.03)	0.427	673/1531 (44.0)	0.97 (0.91, 1.04)	0.420
**Total**	8973/13,583 (66.1)	―		5609/13,033 (43.0)	―	

Abbreviations: HIV, human immunodeficiency virus; aPR, adjusted prevalence ratio; CDMX/EdoMex, Ciudad de México and Estado de México

^†^Adjusted prevalence ratios (aPR) and their 95% confidence intervals (CI) were obtained via Poisson regression models for each outcome that invoked robust standard errors and adjusted for online recruitment venue, age, education, insurance type

^‡^ A total of 13,583 reported HIV negative/unknown status. Denominators for each outcome and region are reported due to missing data.

In adjusted analyses, participants residing in Noroeste (aPR (95% CI) = 0.94 (0.90, 0.98)), Noreste (aPR (95% CI) = 0.94 (0.90, 0.99)), Centro (aPR (95% CI) = 0.94 (0.90, 0.98)), and Bajío/Occidente (aPR (95% CI) = 0.94 (0.91, 0.98)) were significantly less likely to have ever received an HIV test than participants residing in CDMX/EdoMex. Moreover, participants residing in Noroeste (aPR (95% CI) = 0.86 (0.79, 0.93)), Noreste (aPR (95% CI) = 0.91 (0.85, 0.98)), Centro (aPR (95% CI) = 0.85 (0.80, 0.92)), and Bajío/Occidente (aPR (95% CI) = 0.88 (0.83, 0.94)) were significantly less likely to have received an HIV test in the past 12 months than participants residing in CDMX/EdoMex (Table 2).

### Findings among participants reporting living with HIV

A total of 1,650 (10.8%) participants reporting living with HIV. Overall, 83.9% reported receiving counseling after HIV diagnosis, 61.9% linked to care < 1 month post-diagnosis, 42.4% received CD4/viral load results < 1 month post-linkage, 38.2% accessed ART < 1 month post-CD4/viral load results, and 87.7% were currently taking ART. Significant variations in timely linkage to care, lab results, ART access, and current ART use by geographical region were observed (Table 3).

**Table 3:** Frequencies and adjusted prevalence ratios of HIV care cascade outcomes by region among HIV-positive men who have sex with men in Mexico—Encuesta de Sexo Entre Hombres, 2017

Region	Received HIV counseling			Linked to care*			Received lab results*			Accessed ART*			Currently on ART		
	n/N^‡^ (%)	aPR† (95% CI)	p-value	n/N^‡^ (%)	aPR† (95% CI)	p-value	n/N^‡^ (%)	aPR† (95% CI)	p-value	n/N^‡^ (%)	aPR† (95% CI)	p-value	n/N^‡^ (%)	aPR† (95% CI)	p-value
Noroeste	95/110 (86.4)	1.04 (0.95, 1.13)	0.415	71/112 (63.4)	0.95 (0.81, 1.11)	0.494	36/109 (33.0)	**0.65** **(0.48, 0.87)**	**0.004**	40/111 (36.0)	0.95 (0.72, 1.26)	0.723	95/112 (84.8)	0.96 (0.88, 1.04)	0.277
Noreste	94/108 (87.0)	1.03 (0.94, 1.12)	0.535	71/110 (64.5)	0.98 (0.84, 1.15)	0.832	32/109 (29.4)	**0.58** **(0.42, 0.78)**	**< 0.001**	57/109 (52.3)	**1.38** **(1.11, 1.72)**	**0.004**	97/110 (88.2)	0.96 (0.89, 1.03)	0.274
CDMX/ EdoMex	528/619 (85.3)	REF	REF	410/624 (65.7)	REF	REF	331/614 (53.9)	REF	REF	222/620 (35.8)	REF	REF	571/626 (91.2)	REF	REF
Centro	194/236 (82.2)	0.98 (0.92, 1.05)	0.563	139/239 (58.2)	**0.86** **(0.76, 0.98)**	**0.021**	84/238 (35.3)	**0.64** **(0.53, 0.79)**	**< 0.001**	83/238 (34.9)	0.91 (0.73, 1.13)	0.376	196/240 (81.7)	**0.91** **(0.86, 0.97)**	**0.006**
Bajío/ Occidente	169/211 (80.1)	0.97 (0.90, 1.05)	0.438	124/210 (59.0)	0.91 (0.80, 1.03)	0.149	78/210 (37.1)	**0.70** **(0.57, 0.86)**	**< 0.001**	83/209 (39.7)	1.04 (0.84, 1.28)	0.749	182/211 (86.3)	0.95 (0.89, 1.01)	0.097
Sur/ Sureste	146/177 (82.5)	1.00 (0.92, 1.08)	0.925	98/181 (54.1)	**0.78** **(0.67, 0.91)**	**0.002**	57/179 (31.8)	**0.60** **(0.47, 0.77)**	**< 0.001**	76/181 (42.0)	1.12 (0.90, 1.39)	0.305	158/182 (86.8)	0.96 (0.90, 1.02)	0.184
**Total**	1226/1461 (83.9)	―	―	913/1476 (61.9)	―	―	618/1459 (42.4)	―	―	561/1468 (38.2)	―	―	1299/1481 (87.7)	―	―

Abbreviations: HIV, human immunodeficiency virus; ART, antiretroviral therapy; aPR, adjusted prevalence ratio; CDMX/EdoMex, Ciudad de México and Estado de México

*Within 1 month of previous step

^†^Adjusted prevalence ratios (aPR) and their 95% confidence intervals (CI) were obtained via Poisson regression models for each outcome that invoked robust standard errors and adjusted for online recruitment venue, age, education, insurance type, year of HIV diagnosis

^‡^ A total of 1,650 reported HIV positive status. Denominators for each outcome and region are reported due to missing data.

**BOLD** p < 0.05

In adjusted analyses, participants residing in Centro (aPR (95% CI) = 0.86 (0.76, 0.98)) and Sur/Sureste (aPR (95% CI) = 0.78 (0.67, 0.91)) were significantly less likely to report timely linkage to care compared with participants residing in CDMX/EdoMex. Those residing in Noroeste (aPR (95% CI) = 0.65 (0.48, 0.87); p = 0.004), Noreste (aPR (95% CI) = 0.58 (0.42, 0.78)), Centro (aPR (95% CI) = 0.64 (0.53, 0.79)), Bajío/Occidente (aPR (95% CI) = 0.70 (0.57, 0.86)), and Sur/Sureste (aPR (95% CI) = 0.60 (0.47, 0.77)) were significantly less likely to report timely receipt of CD4/viral load results than those residing in CDMX/EdoMex. MSM in Noreste (aPR (95% CI) = 1.38 (1.11, 1.72)) were significantly more likely to report timely access to ART than those residing in CDMX/EdoMex. MSM in Centro (aPR (95% CI) = 0.91 (0.86, 0.97)) were significantly less likely to be currently taking ART compared with participants residing in CDMX/EdoMex (Table 3).

**Fig. 1:**
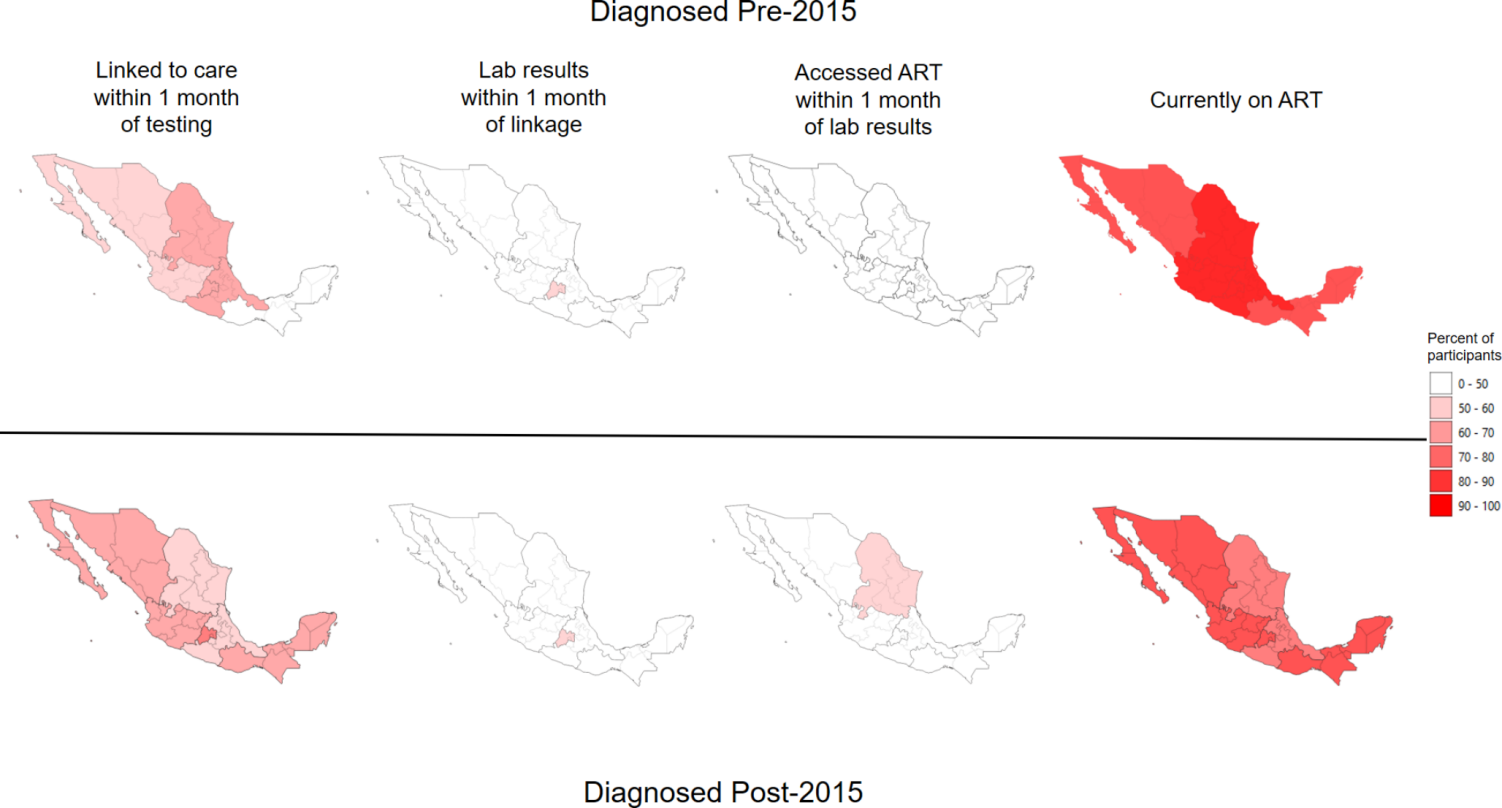
Percent of MSM participants reporting HIV-positive status with selected HIV care cascade outcomes by geographic region and stratified by pre/post 2015 HIV diagnosis—Encuesta de Sexo Entre Hombres, Mexico, 2017

About half of participants living with HIV (51.0%) had HIV diagnosed before the 2015 immediate ART initiation policy (Table 1). For all regions, < 60% of respondents reported receiving timely lab results and timely ART access. Participants with HIV diagnosed in 2015 or after had marginally greater proportions obtaining timely access to ART compared to those with HIV diagnosed before 2015. Yet, participants with HIV diagnosed in 2015 or after had a lower proportion currently taking ART compared with those with HIV diagnosed prior to 2015 in all regions (Fig. 1; Table 4).

**Table 4:** Frequencies and percentages of HIV care cascade outcomes by region among men who have sex with men in Mexico with HIV diagnosed pre- and post-2015—Encuesta de Sexo Entre Hombres, 2017

Region	Among participants reporting HIV-positive status					
	**Received HIV counseling**	Linked to care*	Received lab results*	Accessed ART*	**Currently on ART**	
	n (%)	n (%)	n (%)	n (%)	n (%)	
Noroeste						
Pre-2015	39 (81.3)	28 (58.3)	16 (34.0)	16 (34.0)	43 (89.6)	
Post-2015^†^	52 (91.2)	40 (69.0)	19 (33.9)	23 (39.7)	48 (82.8)	
Noreste						
Pre-2015	52 (89.7)	40 (69.0)	16 (27.6)	27 (46.6)	55 (96.5)	
Post-2015^†^	41 (83.7)	30 (58.8)	16 (32.0)	29 (58.0)	40 (78.4)	
CDMX/EdoMex						
Pre-2015	280 (84.3)	204 (60.7)	173 (52.3)	109 (32.4)	316 (94.3)	
Post-2015^†^	245 (86.9)	203 (71.7)	156 (56.1)	111 (39.8)	249 (87.4)	
Centro						
Pre-2015	85 (85.9)	67 (65.7)	42 (41.2)	35 (34.3)	94 (92.2)	
Post-2015^†^	108 (79.4)	71 (52.2)	41 (30.4)	48 (35.6)	101 (73.7)	
Bajío/Occidente						
Pre-2015	82 (76.6)	62 (57.9)	42 (39.3)	37 (34.9)	100 (92.6)	
Post-2015^†^	86 (84.3)	61 (60.4)	35 (34.7)	45 (44.6)	81 (80.2)	
Sur/Sureste						
Pre-2015	73 (83.0)	42 (47.2)	27 (30.7)	35 (38.9)	79 (87.8)	
Post-2015^†^	73 (82.0)	56 (60.9)	30 (33.0)	41 (45.1)	79 (85.9)	
**All**						
Pre-2015	611 (93.5)	443 (59.9)	316 (43.1)	259 (35.1)	687 (92.8)	
Post-2015^†^	605 (84.6)	461 (63.9)	297 (41.8)	297 (41.6)	598 (82.6)	

Abbreviations: HIV, human immunodeficiency virus; ART, antiretroviral therapy; CDMX/EdoMex, Ciudad de México and Estado de México

*Within 1 month of previous step

^†^Post-2015 includes year 2015 and thereafter

## Discussion

Understanding geographical variations in HIV care can help inform distribution of resources and intervention implementation focused on achieving 90/90/90 among MSM in Mexico, which is one step in reaching broader 90/90/90 aims in Latin America. In the ESEH study, significant geographical differences in the HIV care continuum were found among a large sample of MSM in Mexico.

One major finding from this study was the geographic variation of ever HIV testing and testing in the past 12 months among HIV-negative/unknown participants. Per the CENSIDA recommendation, MSM should be tested at least every 12 months, and in some cases more frequent screening is recommended, such as every 3 months. [27] In our sample, a third had never had an HIV test (33.9%), similar to previous national CENSIDA figures in 2017 (39.8%).^(28)^ In comparison to other research estimating ever and recent testing in Tijuana among MSM (63.5% and 36.8%, respectively), [12] our regional Noroeste estimates, where Tijuana is located, were similar (64.3% and 39.8%, respectively). The lowest proportion of recent HIV tests occurred in Centro (39.6%) and the highest proportion occurred in CDMX/EdoMex (47.3%). The CDMX/EdoMex region may have higher prevalence of testing than other regions because of more resources per capita, decreased HIV- and gay-related stigma, and better accessibility (e.g., testing hours/location, transportation, etc.), and is the location of the largest HIV clinic in the country. [28] Geographic variation of HIV testing, centered primarily in urban areas, is similar to previous research in Latin America. [29, 30]

The lack of recent HIV testing in most regions of the country is concerning given testing is the first step to prevention or care continuums. [31] With the expanding availability of PrEP in Mexico, adopting a status-neutral approach to HIV counseling and linkage to follow-up services could benefit both prevention and care outcomes among MSM. The status-neutral approach begins with HIV testing, and motivates individuals to know their status and then initiate the same approach for engagement in supportive services regardless of HIV status. [31] This type of approach could motivate HIV-negative MSM to test and test routinely as part of their engagement in a prevention strategy, including PrEP continuity. Furthermore, strategies such as HIV self-testing have been shown to reduce traditional barriers to testing (e.g. transportation, stigma, etc.) and engage people not traditionally reached by current HIV testing efforts and could be considered as a supplemental strategy to increase testing among MSM. [7] Additionally, further research should aim to identify any specific regional barriers that could be stalling routine HIV testing rates.

Although it is encouraging that current ART use was above 80% in all geographic regions, timely progression through the HIV continuum of care was low and we did find geographic variations in care continuum outcomes among PLWH. In Latin America linkage to care data are scarce, though unpublished research in Venezuela and Colombia suggest similar suboptimal linkage to care (40% and 60%, respectively). [32] In our sample, only 61.9% were linked to care within a month of HIV diagnosis, with the highest proportion in CDMX/EdoMex (65.7%) and the lowest proportion in Sur/Sureste (54.1%). Poor linkage to care could be due, at least in part, to a lack of healthcare access and a fragmented healthcare system in Mexico. The Mexican health system is stratified into 3 main providers: (1) social security (IMSS, ISSSTE, PEMEX, SEDENA, MARINA) used by those employed, their families, and retirees, (2) public federal health insurance (National Commission of Social Health Protection, Seguro Popular (now INSABI), Bienestar) used by those self-employed and unemployed, and (3) private insurance. [21] Previous research has indicated that 48.5% of the Mexican population may lack effective access to healthcare services, [33] and this may be worse for stigmatized groups like PLWH. [34, 35] Moreover, although IMSS and Seguro Popular cover 93% of PLWH on ART in Mexico, these systems have limited data sharing agreements and have differences in their ART use guidelines, making switching health systems difficult. [36] Further, with recent changes of Seguro Popular to INSABI in the context of the COVID-19 pandemic, a budget drop of 36.9% for specialized conditions (including HIV) occurred between 2020 and 2021, affecting the number of patients for whom the system can provide care. [37] Access to healthcare and health insurance coverage are key components to navigating linkage to HIV care and downstream care outcomes. Although we controlled for insurance type in the multivariate analysis, it is possible that delays in enrollment or switching insurance systems was not fully captured yet could have impacted timely linkage. Strategies to assist uninsured persons to obtain insurance coverage, decrease administrative burdens within and between healthcare systems, and increase funding for specialized conditions may be useful to improving linkage to care, particularly for regions like Centro and Sur/Sureste where timely linkage to care lags. Further, we did observe regional variation in insurance status in our sample, therefore these impacts may be more acutely experienced in regions such as Centro and Sur/Sureste that had the highest proportions of participants with either no insurance or covered by Seguro Popular (now INSABI); future analyses could assess potential heterogeneity in associations between insurance providers and HIV care outcomes by region to better elucidate the potential impact of systems on care in different regions. Additional research could also consider how health system navigation and transfers between HIV care systems could impact linkage and care outcomes.

Another salient finding of our study was that only 42.4% of HIV-positive participants had received lab results within 1 month following linkage to care; all regions had significantly lower proportions of MSM who had received labs in a timely manner compared to the CDMX/EdoMex region, and Noreste had the lowest proportion (29.4%). A key explanation for this finding could be a limited number of regional HIV service providers approved by the General Director of Quality and Education on Health and the Commission formed by the Social Protection in Health and State Health Secretariats (SPSS). Based on a 2018 report from SPSS, there were a total of 102 accredited federally approved HIV services providers. [38] CDMX/EdoMex far surpassed the national average of 0.52 federally approved HIV clinics per 10,000 km^2^ with 6.71 federally approved HIV clinics per 10,000 km^2^_,_ [38, 39] while Noreste only had 0.32 federally approved HIV clinics per 10,000 km^2^. [38, 39] Though the proportion of PLWH in CDMX/EdoMex is higher than all other regions (13.2%), the limited distribution of federally approved HIV clinics in other regions suggests that improving this distribution could be critically important to overcome accessibility barriers (e.g. existence, distance, time of transportation, etc.) and improve successful progression through the care continuum. [40, 41] Additionally, challenges in the state public procurement processes for laboratory testing equipment and resources could have affected the timely access to CD4 and viral load tests. These systematic barriers of lab resources and processing, funding, and HIV clinic and laboratory distribution may hinder progress on care continuum outcomes; these will need to be addressed to improve care outcomes and reduce regional disparities in HIV care among MSM in Mexico.

Our study found greater proportions of those with HIV diagnosed in 2015 or later having obtained timely access to ART in all regions, in comparison to those with HIV diagnosed pre-2015. This finding could signify that the 2015 implementation of policy that indicated everyone living with HIV should initiate ART regardless of CD4 cell count, [22, 23] is improving timely access and initiation of ART across all of Mexico. Interestingly, however, those with HIV diagnosed in 2015 or later had smaller proportions currently taking ART in all regions. This finding could be explained by differences in age. Previous literature has found that those with older age have greater success of ART adherence. [42, 43] Additionally, this finding could be partially explained by lack of perceived need for ART due to recency of diagnosis, [10] though recent research has suggested that those with recent HIV diagnosis may be more ready for ART adherence. [44] Future strategies to support test-and-treat policies could focus on promoting the importance of early initiation of ART and improving sustained ART use in all regions and other Latin American countries, [32] particularly among younger MSM and MSM with recently diagnosed HIV.

### Limitations & Strengths

First, all items were self-reported and subject to response bias. Second, this study was cross-sectional meaning temporal differences could not be determined. Third, constructs like nativity and access to transportation, were not collected and therefore could not be included in adjusted analyses. Fourth, viral suppression among MSM living with HIV was not assessed. Fifth, items assessing the PrEP continuum of care were not included. Finally, the results of this study may not be generalizable to MSM who do not have access to the internet. Despite the limitations, several estimates from this study were similar to those of previous national and regional research. Further, this study incorporated a large and geographically diverse sample of MSM in Mexico to describe care outcomes in regions that have been previously understudied.

## Conclusion

Critical work needs to be done for Mexico to reach the 90/90/90 goals, particularly among MSM who are disproportionately affected by HIV. Significant regional differences in the HIV care continuum were identified in this study and these disparities may hinder achievement of national goals. Understanding geographical variations in HIV care for MSM in Mexico is one important step to inform efforts for ending HIV/AIDS by 2030 in Latin America. Resource allocation and interventions tailored to region-specific needs should be considered in future care and prevention efforts.

## Data Availability

Data may be made available with request to the corresponding author.

## Code Availability

Code used to run the analyses for the purposes of this manuscript may be made available with request to the corresponding author.
